# *ТР53* Codon 72 Polymorphism and Human Papilloma Virus-Associated Cervical Cancer in Kyrgyz Women 

**DOI:** 10.31557/APJCP.2019.20.4.1057

**Published:** 2019

**Authors:** Jainagul Isakova, Denis Vinnikov, Nurbek Bukuev, Elnura Talaibekova, Nazira Aldasheva

**Affiliations:** 1 *Institute of Molecular Biology and Medicine, *; 3 *National Center of Oncology and Hematology, Bishkek,*; 2 *School of Public Health, Al-Farabi Kazakh National University, Almaty, Kazakhstan. *

**Keywords:** *ТР53* gene, Arg72Pro, polymorphism, cervical cancer, Kyrgyz population

## Abstract

**Background:**

The aim of this study was to ascertain the magnitude of association of gene *ТР53* Arg72Pro polymorphic marker with cervical cancer (CC) in Kyrgyz women.

**Methods:**

We identified and included 205 women of Kyrgyz ethnicity for this case-control study, of whom N=103 were women (mean age 53.5 ± 10.0 years) with histologically confirmed CC and N=102 controls (mean age 46.5 ± 8.5 years). We detected human papilloma virus (HPV) DNA types 16 and 18 using polymerase chain reaction (PCR) with hybridization/fluorescent detection. Genotypes of *ТР53* gene Arg72Pro polymorphism were identified using PCR-RFLP assay.

**Results:**

Eighty-eight percent (90/103) women with CC had HPV, of whom 43.4% (39/90) had HPV type 16, 24.4% (22/90) had HPV type 18, whereas 32.2% (29/90) carried both types. The univariate analysis of allele and genotype distribution of Arg72Pro polymorphic marker of* ТР53* gene showed no difference between CC and control groups (χ^2^=1.24, р=0.54). However, when CC cases associated with HPV were tested against controls, Arg72 allele and Arg72Arg genotype prevalence were greater compared to controls (χ²=7.25; р=0.027 for genotypes and χ²=6.83; р=0.009 for alleles). In HPV-positive women, Arg72Arg genotype of *ТР53* gene was associated with a 1.85-fold increase in the likelihood of CC (OR=1.85 [95% confidence interval (CI) 1.03-3.32]), whereas Arg72 allele increased this likelihood 1.94-fold (OR=1.94 [95% CI 1.20-3.15]).

**Conclusions:**

Arg72Arg genotype and Arg72 allele of *ТР53* gene in Kyrgyz women increase the risk of HPV-associated CC.

## Introduction

Cervical cancer (CC) incidence across the globe varied dramatically from somewhat 3.5 to 21 cases per 100 thousand women (GLOBOCAN Cancer Fact Sheets: Cervical cancer). In developed countries, this cancer incidence shows the trend for reduction, which may associated with better awareness of risk factors, active prevention and advanced pre-cancer diseases recognition and treatment. On the contrary, in developing world, CC is the leading type of cancer among all malignant tumors in women. Some small, but persistent CC incidence increase is reported in Kyrgyzstan since mid-90s. In Kyrgyzstan, CC incidence in 2011 was 11.0 cases per 100,000, including 60.5% with stage I–II; 30.6% with stage III and 8.9% with stage IV. Mortality rate was 3.3 per 100,000, one-year mortality was 46.9%; and five-year survival was 39.7%. The mean age of CC patients was 40 years (Izmailova and Makimbetov, 2014). 

CC prevention is one of prioritized directions in public health and health promotion. CC is to large extent associated with human papilloma virus (HPV). HPV DNA was found in more than 99.5% of biopsy material of CC patients (Dijkstra et al., 2014). At present, there exist more than 120 HPV types, whereas 30 of those infect anogenital area. Some HPV types exhibit high oncogenic risk including types 16, 18, 31, 33, 35, 39, 45, 51, 52, 56, 58, 59, 66, 68, 73 and 82. HPV type 16 is considered as most prevalent in CC and found in up to 50-70% CC cases, followed by type 18 (confirmed in 10-20% CC cases) (Arbyn et al., 2014; Bruni et al., 2015). Vaccination against HPV was initiated in 2007 in countries with efficient public health system. On the contrary, such vaccine is not yet available in low-income countries such as in Kyrgyzstan.

Usually it takes ten years and more for malignant transformation from the initial contamination. Viral genome may remain latent for a very long time, and its activation is one of the pivotal stages in oncogenesis, however, this process remains poorly understood. Recent studies have shown that aside from HPV, suppressor genes may also play role in CC pathogenesis. р53 gene is one of the few most studied suppressor genes, important for induction and progression of carcinogenesis associated with HPV (Arbel-Alon et al., 2002; Brenna et al., 2004). Few studies have confirmed allele status of Р53 gene is associated with CC in HPV-positive women (Ciotti et al., 2006; Gudleviciene et al., 2006; Katiyar et al., 2003). 

The aim of this study was to assess the role of Arg72Pro polymorphic locus of *ТР53* (*rs1042522*) gene in CC of HPV-positive women in Kyrgyz Republic. This work is the first genetic study of HPV in Kyrgyz Republic. 

## Materials and Methods

*Study Outline and Sample Population*

This study was designed as case-control. Cases were 103 CC women, treated in the in-patient department of gynecology of the National Center of Oncology and Hematology in Bishkek from 2015 to 2016. In all included cases the CC diagnosis was based on morphology tests, clinical and ancillary examinations, including ultrasonography and computer tomography (CT), oncogynecologist visit, smear cytology using Papanikolau method and colposcopy. In all cases the diagnosis was verified with biopsy of smears obtained at gynecological consultation and after surgery.

One-hundred and two more women were controls, who were unrelated with the women in the main group. Women with the history of any cancer were excluded from controls at a stage of screening. The mean age of CC women was 53.5 ± 10.0 years and 46.5 ± 8.5 years in controls. Only women of Kyrgyz ethnicity constantly residing on the territory of Kyrgyz Republic were included in cases and controls. All participants of both groups signed informed consent to participate. The study protocol was approved by the local institutional review board of the Institute of Molecular Biology and Medicine in Bishkek Kyrgyz Republic (Minutes #3, dated 03.01.2014).

*Molecular methods of cervical smears tests*

Epithelial cell smears from the cervix were the material for molecular tests in this study. High carcinogenic risk HPV type 16 and 18 were detected and differentiated using reagents set «AmpliSense, HPV 16/18-FRTA» via PCR with hybridization/fluorescent detection in real time. This method is based on a simultaneous HPV DNA segments amplification along with the segment of β-globin gene DNA, using for the purpose of endogenous internal control. All test stages, such as nucleic acid separation, amplification, analysis and interpretation, were completed as of the producer’s manual (High-Risk Human Papilloma virus Infections). 

*DNA extraction*

DNA was extracted from the white cells of peripheral blood, using the standard method of phenol-chloroform extraction. 

*Determination of ТР53 Genotype*

**Figure 1:**
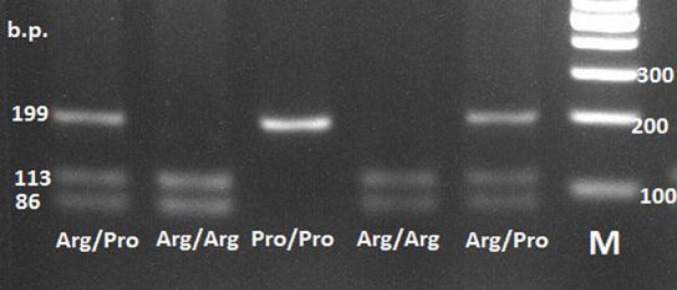
Electrophoretic Picture of *ТР53* Gene Arg72Pro Polymorphic Locus in 3% Agarose Gel after Processing with BstUI Endonuclease. Pro/Pro genotype was 199 b.p. long; Arg/Pro genotype 199, 113 and 86 b.p. long; Arg/Arg 113 and 86 b.p. long. М is a DNA molecular mass marker 100 b.p. long

We genotyped single-nucleotide Arg72Pro polymorphism in the fourth exone of *ТР53* gene using PCR with subsequent restriction of its products and restriction fragments length polymorphism (PCR-RFLT) assay. PCR was conducted in Hybaid (UK) amplifier. We amplified *ТР53* gene Arg72Pro locus with direct 5`TTGCCGTCCCAAGCAATGGATGA–3` and reverse 5`TCTGGGAAGGGACAGAAGATGAC–3` primers. Twenty µL of amplification mixture contained 1x buffer for Go-Taq-polymerase, 1.5 мМ MgCl_2_, 1.6 мМ of dNTP mixture, 5 pmol of each primer, 100 nanogram of genome DNA and 1 unit of Go-Taq- polymerase (Promega, USA). Amplification protocol included preliminary denaturation at 94°С for 5 minutes with 35 subsequent cycles of denaturation at 94°С (30 sec), annealing at 60°С (30 sec) and chain elongation at 72°С for 30 sec. The program was finalized with elongation at 72°С for 5 min. Amplification products were then hydrolyzed with BstUI endonuclease (Promega, USA) at 37о for 16 hours. Restriction products were separated usng elecroforesis in 3% agarose gel and the fragments were visualized by means of GelDoc IT system (UVP, Great Britain) in ultraviolet light. Arg72 allele has fragments 133 and 86 b.p. long, where as 72Pro minor allele is 199 b.p. long (Figure 1).

*Statistical analysis*

We used previous report from Chinese population to calculate the current study sample size using the built-in formula in Equation 6.10. In the preceding study (Ye et al., 2010), the odds ratio (OR) of CC in Arg72Pro carriers was 3.83. Given the number of exposed controls in our study 35%, case:control ratio 1:1 and Z-alpha 1.96, the minimum required number of cases for OR 3.83 should be 36 with the corresponding number of controls 36 also to attain 80% power. Taking stricter OR 2.38 for Pro72Pro from the same study, the minimum required number of cases would be 86. Therefore, our study sample size of 102 cases ensures sufficient study power (100% and 84% correspondingly). We analyzed data in GraphPad Prism v 5.04. We tested the observed distributions vs. the expected Hardy-Weinberg equilibrium via χ^2^. Genotype distributions were analyzed in 2*2 tables and χ^2^ criterion with Yates correction, whereas the associations were expressed as OR with their corresponding 95% confidence intervals (CI). Differences were considered significant with р<0.05.

## Results

*Subjects’ profile*

At the time of diagnosis, the age of CC patients ranged from 20 to 77 years. Forty-five women (44%) in the CC group (N=103) were in the age range from 51 to 60 years, whereas younger women with the age range 41 to 50 years were less prevalent (26.4 %, N=27). There were very patients in the age group of 20 to 30 years (N=2, 2%), 15 patients (14.5%) were 31-40 лет years old, 9 women (8.9%) were 61-70 years old and only 5 patients (5%) were older than 70. 

Among the range of existing CC stage classifications, we used FIGO scale to grade patients. In the current sample, clinical stage Ι was found in 3 patients (3%), stage ΙΙ in 35 patients (34%), stage ΙΙΙ was identified in 62 cases (60%), and stage ΙV was found in 3 (3%) patients. Squamous cancer was diagnosed in 98 (95%) women, whereas adenocarcinoma was found in 5 (5%) patients. G2 tumor was verified in 100 (97%) included women, whereas G3 in 3 (3%).

**Table 1:** The Distribution of Genotypes and Alleles of Arg72Pro Polymorphism of ТР53 Ggene in HPV-Positive Women with CC Compared to Controls

Polymorphism	Genotypes and alleles	HPV-positive CC, N (%)	Controls, N (%)	OR (CI 95%)
Arg72Pro of TP53 gene (rs1042522)	Arg72Arg	60 (67)	53 (52)	1.85 (1.03-3.32)
	Arg72Pro	27 (30)	36 (35)	0.79 (0.43-1.44)
	Pro72Pro	3 (3)	13 (13)	0.24 (0.06-0.86)
	Arg72	147 (82)	142 (70)	1.94 (1.20-3.15)
	72Pro	33 (18)	62 (30)	0.51 (0.32-0.83)

HPV, human papilloma virus; CC, cervical cancer; OR, odds ratio; CI, confidence interval

**Table 2:** Comparative Analysis of Genotyping Outcomes of Arg72Pro Polymorphic Locus of *TP53* Ggene with Clinical and Morphological Tumor Attributes

Grades	Arg72Arg N (%)	Arg72Pro N (%)	Pro72Pro N (%)	Р-value
Grade of differentiation				
G2	59 (98)	33 (97)	8 (88)	NS
G3	1 (2)	1 (3)	1 (11)	
FIGO stage				
I	1 (2)	1(3)	1 (11)	
II	26 (43)	7 (20)	2 (22)	NS
III	31 (52)	26 (76)	5 (55)	NS
IV	2 (3)	-	1 (11)	
Histological type				
Squamous	57 (95)	33 (97)	8 (88)	NS
Adenocarcinoma	3 (5)	1 (3)	1 (12)	NS

FIGO, International Federation of Gynecology and Obstetrics; NS, non-significant

Ninety women (87%) of the entire pool of 103 CC patients were infected with HPV, of whom 43.4% (39/90) had HPV type 16; 24.4% (22/90) had type 18 and 32.2% (29/90) exhibited HPV of both types (16/18). HPV was found in one subject of the control group. 

*Distribution of genotypes and allele frequencies among subjects*

Genotype distribution of gene *ТР53* Arg72Pro polymorphic locus in CC women and in control group reflected Hardy-Weinberg equilibrium (χ^2^=0.019; р=0.88 in CC group and χ^2^=2.80; р=0.093 in controls). In both group we found that Arg allele and Arg72Arg genotype prevailed over the alternative variants. Arg72Arg, Arg72Pro and Pro72Pro genotype prevalence in the group of women with CC were 58%, 33% and 9% correspondingly, whereas Arg and Pro alleles’ frequency was 0.74 and 0.26.

The corresponding frequency of Arg72Arg, Arg72Pro and Pro72Pro genotypes in the control group was 52%, 35% and 13%, whereas Arg and Pro allele’ frequency was 0.70 and 0.30. We did not find significant differences in the prevalence of genotypes and alleles of gene Р53 Arg72Pro polymorphic locus when comparing CC patients with controls (χ^2^ = 1.24, р=0.54).

*Genotype analysis on HPV-positive CC*

Given that CC with or without HPV may be associated with some genetic input, we also analyzed the frequency of allele and genotype carriage in both CC women with HPV and controls. HPV-positive women with CC showed statistically significant higher prevalence of Arg72 allele and Arg72Arg homozygous genotype when compared to controls (χ²=7.25; р=0.027 for genotypes and χ²=6.83; р=0.009 for alleles) (Table 1). In HPV-positive women with Arg72Arg genotype, we found a 1.85-fold increase (OR=1.85 [1.03-3.32]) of CC risk, whereas such risk was 1.94 when Arg72 allele was present (OR=1.94 [1.20-3.15]). 

*The association of CC stage and differentiation with the genotypes of TP53 gene Arg72Pro polymorphic locus*

Patients were divided into three groups as of their genotypes. The first group consisted of patients with Arg72Arg homozygous genotype; the second group was formed from Arg72Pro heterozygous genotype; finally, Pro72Pro minor genotype made the third group (Table 2).

*The association of genotype with tumor malignancy (G)*

In our sample, more than half of all CC had II degree of differentiation. Moderate differentiation (G2) was present in 97% cases (n=100), whereas low differentiation (G3) was identified in 3% (n=3) (Table 2). As Table 2 shows, we could not identify the association of Arg72Pro genotype polymorphisms with the degree of tumor malignancy. 

*The association of genotype with the disease stage (FIGO)*

We also analyzed the distribution of tumor stages in a sample of CC women. Clinical stage Ι was found in 3 patient (3%), stage ΙΙ in 35 patients (34%), stage ΙΙΙ was identified in 62 cases (60%), and stage ΙV was found in 3 (3%) patients. In a comparative analysis, we could not associate the stage with alleles or genotypes of P53 gene’s Arg72Pro polymorphic locus (Table 2).

*The association of genotype with tumor histological type*

We also tested the association of genotypes of TP53 gene’s Arg72Pro polymorphic locus with tumor histological types. Histological examination revealed squamous cancer in 97% women with CC, whereas adenocarcinoma was found in 3%. There was no statistically significant difference in the distribution of squamous cancer or adenocarcinoma in various genotype combinations (Arg72Arg, Arg72Pro or Pro72Pro) (Table 2).

## Discussion

CC is believed to be a HPV-associated disease (Arbyn et al., 2014), and in our study, 87% (90 out of 103) women with CC had confirmed HPV. Our analysis showed that CC risk in HPV-positive women was associated with Arg allele carriage (OR = 1.94; 95% CI 1.20–3.15) and Arg/Arg genotype (OR = 1.85; 95% CI 1.03–3.32). On the contrary, Pro allele was protective against CC (OR = 0.51; 95% CI 0.32–0.83), as was Pro/Pro genotype (OR = 0.24; 95% CI 0.06–0.86). Our data also confirm the association of Pro72Arg marker of *ТР53* gene with CC in HPV-positive women of Kyrgyz ethnicity. 

*ТР53* gene is located on a short shoulder of the 17th chromosome (17p13.1) (Matlashewski et al., 1987), contains 11 exons and transcribes 2.8 kbmRNA. Phosphopotein р53 is a protein product of the gene and contains 393 amino acids. р53 protein regulates cell cycle, induces apoptosis in case DNA impairment cannot be repaired, and stabilizes genome. Cell cycle regulation impairment, which is caused by р53 activity change, will promote cancer progression. Such defect can be caused both by mutations and polymorphism (Kim et al., 2001).

*ТР53* has around 200 known single nucleotide polymorphisms (SNP) [http://www-p53.iarc.fr/]. Guanine replacement with cytosine in position 215 of the fourth exon (Arg72Pro (rs1042522)) is considered one of the most meaningful polymorphisms of *ТР53* gene. Such mononucleotide replacement results in two versions of р53 protein, including the one with arginine (Arg) and one with proline (Pro) in the 72nd codon (George, 2011). Biochemical properties of these two protein variants differ. Data from the literature show that 72Arg *ТР53* protein may have a greater potency to initiate apoptosis compared to 72Pro *ТР53*, because 72Arg protein penetrates mitochondria quicker, and that entails cytochrome C release into the cell cytoplasm and subsequent apoptosis induction (Dumont et al., 2003).

They also reported that Е6 oncoprotein, coded by HPV-18 and HPV-16, may interact with р53 protein, and that induces its degradation (Thomas et al., 1999). Storey et al. (Storey et al., 1998) found that p53Arg allele is destroyed by Е6 HPV-18 much faster than p53Pro. Mutant variant of this protein loses this property, and that leads to cancer transformation. The fact that all cancer cells exhibit the mutant variant of р53 inclined the researchers to conclude that the normal variant of this protein may act as a carcinogenesis suppressor. Based on that, the authors hypothesized that allele status of *ТР53* gene may promote CC, associated with papilloma virus. 

Storey et al., (1998) found a 7-fold increase of CC risk in HPV-positive women with Arg72Arg genotype. Min-min et al., (2006) also found higher CC risk in HPV-positive Chinese women with Arg72Arg genotype when compared to their Pro72Pro counterparts. Other studies in India (Saranath et al., 2002), South Africa (Pegoraro et al., 2002) and Santiago (Ojeda et al., 2003) also found greater CC risk in HPV-positive women with Arg72Arg genotype. Population-based studies in the European region, including Italy (Ciotti et al., 2006), Lithuania (Gudleviciene et al., 2006) and Germany (Zehbe et al., 2001) identified similar association of higher CC risk in HPV-positive women with Arg72Arg genotype when compared to two other variants. On the contrary, other authors reported no association of Arg72Pro polymorphic locus with CC risk in HPV-positive women (Malcolm et al., 2000; Nishikawa et al., 2000; Rosenthal et al., 1998). Our findings are quite consistent with a number of other studies showing higher susceptibility of HPV-positive women to CC when having Arg72Arg genotype and 72Arg allele. 

Of note, the prevalence of *ТР53* gene Arg72Arg polymorphism variants may depend on the geographical location. Thus, Arg72 allele prevalence in the Irish was 0.88; 0.72 in Italians; 0.63 in Germans; 0.65 in Russians; 0.52 in our sample of Kyrgyz; 0.42 in Koreans; 0.44 in Japanese; 0.34 in Jews; 0.33 in South Africans and 0.27 in Indians (Nishikawa et al., 2000; Kim et al., 2001; Arbel-Alon et al., 2002; Pegoraro et al., 2002; Katiyar et al., 2003; Ciotti et al., 2006). In other words, Arg72 allele prevalence lessens closer to the equator. This may be important to verify the distribution and frequency of selected neoplasms associated with HPV 16 and 18 in the given population. 

Ethnicity is an important contributor to allele distribution in the general population since numerous studies have shown different prevalence of specific alleles, including mutations, between ethnic groups. Therefore, selection of ethnically homogenous groups is pivotal for molecular and genetic studies. In this analysis, we report genotyping data of CC patients along with controls from one laboratory and using standardized methodology. The groups were compiled from one population, which prevents inter-population variations.

The strength of this report is the first presentation of the association of Arg72Pro polymorphic locus with CC in HPV-positive women in Kyrgyz population. The limitations of this study are small sample size, inclusion of only one gene for the polymorphic locus evaluation.

In conclusions, given the high prevalence of papilloma viral infection among women combined with HPV etiology in CC, identification of the population with high genetic risk of CC is of significant importance. This will allow reducing such risk, since timely vaccination against HPV will be targeted. 

## Conflict of interest

The authors declare no conflicts of interest. 

## Ethics approval and consent to participate

This study protocol was approved by the local committee on bioethics of the Institute of Molecular Biology and Medicine (Minutes #1, January 14, 2016), Bishkek, Kyrgyz Republic.
